# BMI and WHR Are Reflected in Female Facial Shape and Texture: A Geometric Morphometric Image Analysis

**DOI:** 10.1371/journal.pone.0169336

**Published:** 2017-01-04

**Authors:** Christine Mayer, Sonja Windhager, Katrin Schaefer, Philipp Mitteroecker

**Affiliations:** 1 Department of Theoretical Biology, University of Vienna, Vienna, Austria; 2 Department of Biosciences, CEES & EvoGene, University of Oslo, Blindern, Oslo, Norway; 3 Department of Anthropology, Faculty of Life Sciences, University of Vienna, Vienna, Austria; Medical University of South Carolina, UNITED STATES

## Abstract

Facial markers of body composition are frequently studied in evolutionary psychology and are important in computational and forensic face recognition. We assessed the association of body mass index (BMI) and waist-to-hip ratio (WHR) with facial shape and texture (color pattern) in a sample of young Middle European women by a combination of geometric morphometrics and image analysis. Faces of women with high BMI had a wider and rounder facial outline relative to the size of the eyes and lips, and relatively lower eyebrows. Furthermore, women with high BMI had a brighter and more reddish skin color than women with lower BMI. The same facial features were associated with WHR, even though BMI and WHR were only moderately correlated. Yet BMI was better predictable than WHR from facial attributes. After leave-one-out cross-validation, we were able to predict 25% of variation in BMI and 10% of variation in WHR by facial shape. Facial texture predicted only about 3–10% of variation in BMI and WHR. This indicates that facial shape primarily reflects total fat proportion, rather than the distribution of fat within the body. The association of reddish facial texture in high-BMI women may be mediated by increased blood pressure and superficial blood flow as well as diet. Our study elucidates how geometric morphometric image analysis serves to quantify the effect of biological factors such as BMI and WHR to facial shape and color, which in turn contributes to social perception.

## Introduction

Facial markers of body composition have been of increasing interest to multiple disciplines, such as evolutionary psychology (e.g., as a marker of attractiveness) and computational face recognition. Since body mass and fat distribution are indicators of various health and live-style aspects, facial cues are likely to influence facial perception and can even be important for forensic purposes.

Nutritional conditions are well known to affect the cheeks and the relative width of the lower face (see Wilkinson [1] for a review, Coetzee et al. [2] in Caucasian and African faces, Lee et al. [3] in Korean faces). Increasing BMI leads to a relative widening of the midface and lower face [3–5] as well as reduced eye height and a widening of the nose [3]. Individuals with lower body fat proportion have a more angular face with relatively narrower cheeks and a pointed chin. The visible parts of the eyes are larger relative to overall facial size, the eyebrows higher and more curved, the mouth wider with upturned corners and thinner lips [6]. In a sample of adolescent women, Windhager and colleagues [7] found that body fat accounted for 8.7% of facial shape variation and was highly correlated with BMI (*r* = 0.87).

Also skin coloration is affected by nutrition, various physiological and hormonal parameters, such as oxygenation [8] and estrogen level [9], but also by emotions such as anger and embarrassment [10,11]. In turn, facial skin color influences individual perception and trait attribution. For example, Stephen and colleagues [12,13] found a cross-cultural effect of skin color on perceived men’s attractiveness and health. Löffler et al. [14] demonstrated that obese individuals had significantly increased skin blood flow and reddish skin color as compared to a control group.

Other lines of research focused on the perception or prediction of BMI from faces. For example, using a principal component model, Wolffhechel et al. [15] could predict 23% of variation in BMI by face shape, 27% by face color, and 30% by combining both face shape and color. Some authors related perceived BMI to judgments of female mate value, such as attractiveness and (perceived) health. Instead of analyzing morphological correlates of BMI, they depicted the facial features associated with perceived “facial adiposity” (i.e., high BMI).

Waist-to-hip ratio (WHR) is considered an indicator of fat distribution in the body. Women with a centralized distribution of fat (high WHR) tend to have more of their fat stored in the intra-abdominal region [16]. In a meta-analysis, Vazquez and colleagues [17] reported a correlation coefficient of 0.34 for BMI and WHR. Higher WHR has been associated with a greater psychological vulnerability to stress and reactive cortisol, especially in lean women [18,19]. It has also been shown that the amount of visceral fat (in contrast to subcutaneous fat) is positively correlated with facial (buccal) fat (*r* = 0.5; [20]).

A broad range of methods have been used to investigate facial shape and texture (color pattern), from single distance measurements and local color values to multivariate shape and image analysis approaches. In this study, we assess the relationship of body mass index (BMI) and waist-to-hip ratio (WHR) with both the shape and color pattern of faces in young adult women. We use a combination of geometric morphometrics and image analysis (“geometric morphometric image analysis”, [21]) that allows us to jointly quantify face shape and texture. We explore the average pattern of association of face shape and texture with BMI and WHR, and the degree to which BMI and WHR can be predicted by facial appearance. Since BMI and WHR represent different aspects of body fat distribution and show a relatively low correlation, we investigate if they relate to different aspects of face shape and texture.

## Materials & Methods

### Participants & Data Acquisition

Our sample consisted of 49 standardized photographs of female participants, aged 18–30 years, with a mean age of 22.7 ± 3.1. Data were collected in 2010 at the University of Vienna. Randomly selected students at the biology campus were informed about the project and asked to participate in the study; about two thirds of them volunteered to participate. In a room without daylight, frontal photographs were taken with a digital reflex camera (Olympus E3) with a 113 mm lens positioned at eye height and 3.15 m away from the participants. Studio lights (Hedler Primalux) were used to standardize the light conditions (no flash, ISO 200, aperture F 8.0, shutter speed 1/25). A color checker (X-Rite ColorChecker Passport) was positioned beside the participants, which was used to standardize the color information of the photos in the post-processing. The participants were instructed to keep a neutral facial expression and their heads were adjusted to the Frankfurt horizontal. All participants had their hair tied back and were asked to remove their glasses, piercings, or earrings. Form the 69 imaged female students, 20 were excluded because the photographs did not meet the quality criteria regarding facial orientation and expression, or because critical data was missing in the questionnaires.

Body weight and body height were measured for each participant and used to calculate body mass index (BMI) as body weight divided by the squared body height (kg/m^2^). In our sample, BMI ranged from 17.0 to 35.4. Waist and hip circumference were measured using a measuring tape and used to calculate the waist-hip ratio (WHR), which ranged from 0.66 to 0.82.

All participants reported that their parents were of Central, Northern, or Western European decent. More than 80% had parents of Austrian decent. Ninety percent of the participants have not been to a solarium in the last year, and none of the remaining women went there more than five times. Only two women used self-tanning lotion within the last year, and one woman did not answer this question. Seventy-seven percent of the women reported not to smoke, additional 14% indicated that they smoke less than 5 cigarettes per day. None of the participants consumed alcohol on the day of data collection. We also collected questionnaire data on the menstrual cycle, but sample size was too low for further statistical analyses. The questionnaire used for this study is shown in the Supporting Information (S1).

The research was conducted in accordance with the ethical guidelines of the University of Vienna. Participation in the survey was entirely voluntary and based on written consent. Each participant was informed about the project and the measurement procedure.

### Landmarks

**Fig 1:**
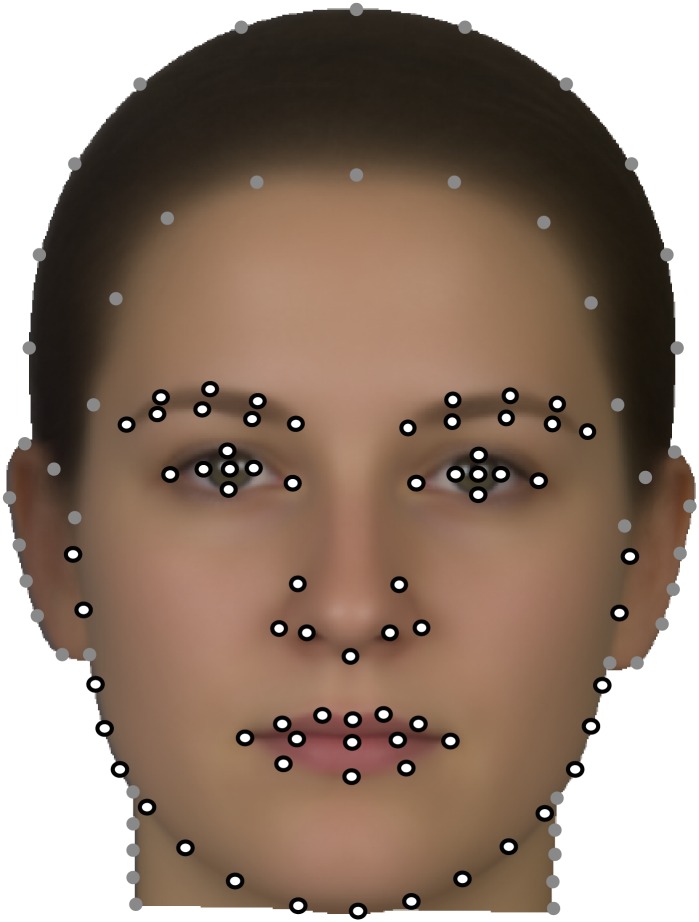
Landmark scheme. The landmarks and semilandmarks used for the shape analysis (black circles) and the image warping (grey disks) are shown on the average face texture deformed to the average face shape.

We recorded 119 anatomical landmarks and semilandmarks (Fig 1) using TPSdig [22]. The basic landmark scheme by Windhager et al. [6] was adapted and extended. We added semilandmarks and four anatomical landmarks on both sides of the neck: two at the height of the chin, and two where the chin outline meets the neck. We also added semilandmarks around the ear, the forehead, and around the vertex to the ears.

Semilandmarks were set in approximately equal distance on the curves. Their exact position was calculated by the sliding landmark algorithm, which allows the landmarks to slide along tangents to the curves in order to minimize overall bending energy, a measure of local shape difference, between each individual and the sample average [23,24]. All of these 119 landmarks and semilandmarks were used for image warping (see below), whereas the shape analyses was based on a subset of 69 (semi)landmarks (Fig 1).

### Shape & Texture Analysis

The landmark configurations were standardized for location, orientation, and scale using Generalized Procrustes Analysis [25,26]. Thereby, the configurations are translated to a common origin, scaled to the same centroid size, and rotated to minimize the sum of the squared distances between the corresponding landmarks. The resulting coordinates—Procrustes shape coordinates—were used to calculate the mean face shape as the average of the shape coordinates. The images were then registered to the resulting mean shape by thin-plate spline interpolation [27] and used to analyze facial texture. Hence, individual face shape was represented by the set of Procrustes shape coordinates, while face texture (the pattern of color across the face) was represented by the RGB values of the images after they were all standardized to the same face shape [28–30]. Individual variation of facial shape and texture were analyzed by principal component analysis (PCA) of the shape coordinates and the pixel RGB values of the registered images, respectively [28,31–34]. Multivariate linear regression was used to analyze the association of facial shape and texture with BMI and WHR. In Mayer et al. [30], we referred to this combined analytic strategy as *geometric morphometric image analysis*.

Several other studies on skin color used the L*a*b* color system [12,35], but we decided for the RGB system because of the geometric independence of the three color values and because a modification of an RGB color value modifies the intensity of the according color but does not lead to *another* color (whereas, e.g., an increase of a* transforms green into red). However, for moderate color variation and R, G, and B all > 0.2, the transformation between RGB and L*a*b* is almost linear, and thus both color systems would lead to very similar average images and regression results.

### Statistical Analysis

The association of BMI with facial appearance was assessed by multivariate linear regression of both shape (shape coordinates) and texture (RGB values of registered images) on BMI. We visualized these associations by adding a multiple of the vector of regression coefficients (corresponding to the predictions for different BMI values) to the mean shape and mean texture. Individual scores of BMI-related facial shape features were computed as projections of the vectors of shape coordinates on the vector of regression coefficients from the regression of shape on BMI. They are equivalent to the scores that would result from a partial least squares analysis of the shape coordinates and BMI. The same approach was used for WHR.

We also predicted BMI and WHR by face shape and texture using conventional multiple regression models. Because such models require substantially more cases than variables, we performed the regressions using different numbers of PCs (instead of all the shape coordinates or all the RGB values) as predictor variables. In addition, we performed regularized regressions (ridge regressions) with all variables and with different values of α, which quantifies the degree of the regularization.

It is well known that such within-sample statistics overestimate the actual out-of-sample prediction accuracy. We thus corrected these estimates by leave-one-out cross-validation, which is asymptotically equivalent to the Akaike information criterion.

All analyses and visualizations were performed using Mathematica 10 (Wolfram Research Inc., Champaign, IL, USA).

## Results

Descriptive statistics for body mass index (BMI) and waist-hip-ratio (WHR) are presented in Table 1. BMI and WHR were moderately correlated (*r* = 0.47). No woman showed severe underweight or severe obesity.

**Table 1:** Descriptive statistics for body mass index (BMI) and waist-to-hip ratio (WHR): mean, standard deviation, range (min-max), and the three quartiles.

	mean	s.d.	range	Q1, Q2, Q3
BMI	23.0	4.0	17.0–35.4	20.3, 21.6, 24.8
WHR	0.72	0.04	0.66–0.82	0.69, 0.72, 0.75

**Fig 2:**
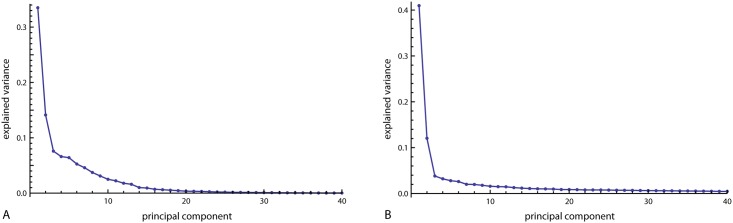
Scree plots. Scree plots for (A) face shape and (B) face texture, showing the fractions of variance accounted for by the corresponding principal components.

**Fig 3:**
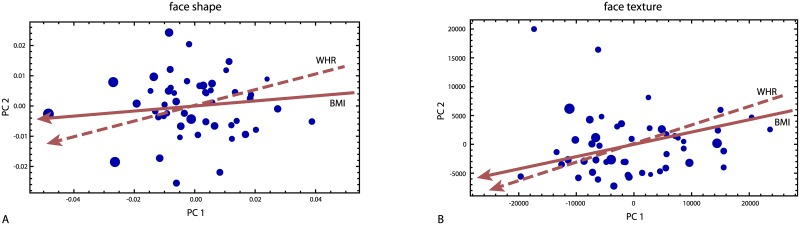
Principal component analyses. Principal component (PC) scores of (A) face shape (landmark shape coordinates) and (B) face texture (RGB values of the standardized images). Each disk represents one individual, where the diameter of the disk is proportional to the individual’s BMI. In (A) the distances between points approximate the shape differences (Procrustes distance) between the corresponding individuals’ faces, whereas in (B) the distances approximate differences in face texture (squared differences in RGB values, summed over all pixels). The solid red lines are the directions in these two-dimensional PC spaces with maximal increase in body mass index (the coefficient vector of the regression of shape on BMI, projected onto PC 1 and 2); the dashed red lines are the directions of maximal increase in waist-to-hip ratio (WHR).

**Fig 4:**
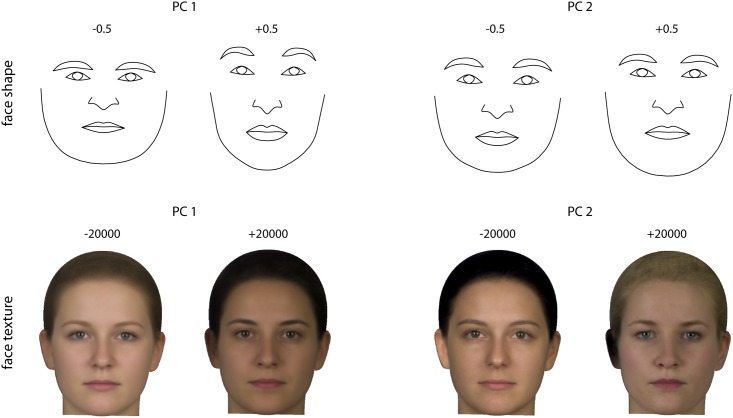
Visualization of the first two principal components (PCs) of face shape (upper panel) and face texture (lower panel). The individual PC scores for these shape and texture features are plotted in Fig 3. The reconstructed faces correspond to a deviation of 0.5 units (Procrustes distance) and 20,000 units from the mean shape and mean texture, respectively, which approximately corresponds to the occurring range of variation along PC1 and to a twofold extrapolation of this range along PC 2.

Facial shape variation was characterized by two dominant principal components (PC), which together accounted for 48% of total variation in the Procrustes shape coordinates (Fig 2A). The scatter plot of the PC scores in Fig 3A demonstrates an association of PC 1 with BMI and WHR: individuals with higher BMI as well as higher WHR tended to have lower scores than individuals with higher BMI (*r* = -0.55 and -0.41 respectively). The PC axes are visualized by reconstructed shapes that correspond to different positions along the axes (Fig 4). The first PC distinguished relatively wide faces with thin lips and low eyebrows from relatively narrow and high faces with larger lips and higher eyebrows. The second PC mainly captured the relative size of the lower jaw.

Variation in face texture was again dominated by the first two PCs, which accounted for 53% of total variation in the RGB values across the standardized face images. The first PC was weakly associated with BMI and WHR (*r* = -0.22 and *r* = -0.28; Fig 3B). The reconstructed textures show that PC 1 represented the overall amount of pigmentation in the skin and hair, while PC 2 contrasted dark-haired and dark-eyed individuals from blond, blue-eyed individuals with a bright and reddish skin color (Fig 4).

The principal components of face shape varied largely independently of the principal components of texture (*R*^2^ < 0.023 for all pairwise comparisons of the first four PCs of shape and texture). Hence, a joint ordination, e.g., via scaled partial least squares [30,36], was similar to the separate ordinations of shape and texture (hence not shown).

**Fig 5:**
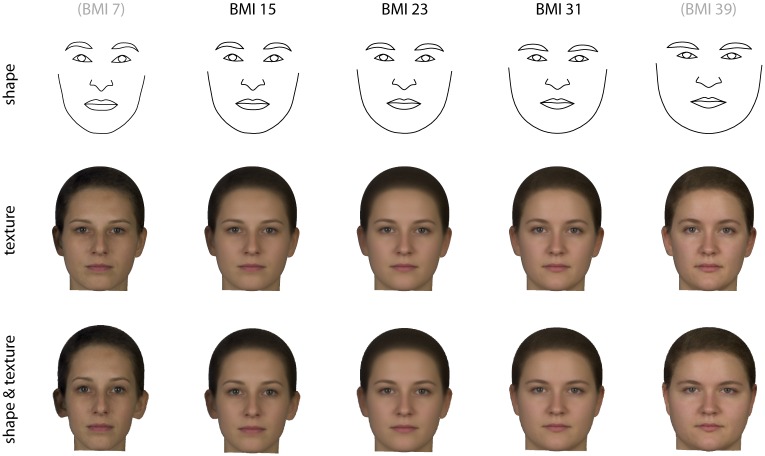
Regressions of shape (upper panel), texture (middle panel), as well as both together (lower panel) on BMI. The reconstructed faces represent the average shapes and textures predicted for BMI 15, 23, and 31, respectively, based on these regressions. These are ±2 standard deviations around the average BMI of 23 and approximately represent the range of variation observed in our sample. The left-most and right-most faces in the figure are twofold extrapolations of this range; they correspond to deviations of ±4 standard deviations from the mean and to (hypothetical) BMIs of 7 and 39.

**Fig 6:**
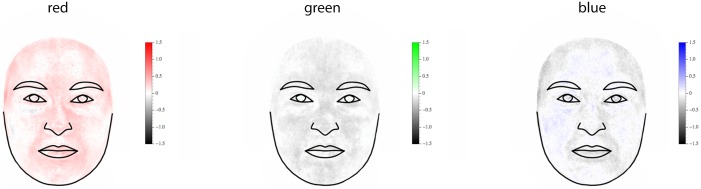
Regression of facial texture on BMI, visualized for each color channel (R, G, B) separately. In contrast to Fig 5, here the RGB values of each pixel were corrected to keep brightness constant (i.e., they were mean-centered separately for each pixel) to focus on the association of hue with BMI.

The association of BMI and facial appearance—assessed via multivariate linear regression of shape and texture on BMI—is visualized in Fig 5. Faces of women with low BMI were narrower and had relatively thicker lips, larger eyes, and higher eyebrows than that of individuals with high BMI, who had a rounder and larger facial outline relative to the size of the eyes and lips. On average, women with high BMI had a brighter and more reddish skin color than women with lower BMI. The multivariate regressions of Procrustes shape coordinates and texture on BMI were significant at *p*<0.001 and *p*<0.002, respectively. In Fig 6 the regression coefficients of the three color channels (R, G, B) on BMI are visualized separately, clearly showing the increase of red values with BMI.

**Fig 7:**
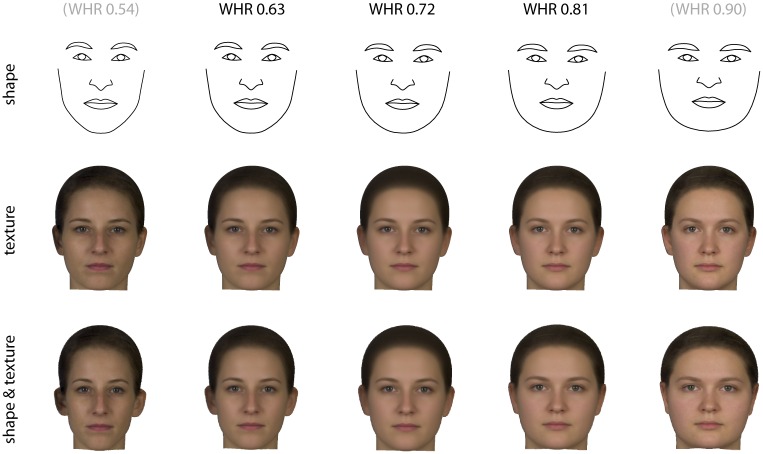
Regressions of shape (upper panel), texture (middle panel), as well as both together (lower panel) on waist-to-hip ratio (WHR). The reconstructed faces represent the average shapes and textures predicted for WHR 0.63, 0.72, and 0.81, respectively, based on these regressions. These are ±2 standard deviations around the average WHR of 0.72 and approximately represent the limits of variation observed in our sample. The left-most and right-most faces in the figure are twofold extrapolations of this range; the correspond to deviations of ±4 standard deviations from the mean and to (hypothetical) WHRs of 0.54 and 0.90.

The association of WHR and facial appearance is visualized in Fig 7. These results were essentially the same as those for BMI in Fig 5: despite the moderate correlation of BMI and WHR, both were associated with the same facial features.

The individual shape scores for the BMI-related face shape features (as shown in the upper panel of Fig 5) had a correlation with BMI of 0.63. The individual scores for BMI-related texture features had a correlation with BMI of 0.35. These correlations indicate that face shape was more closely associated with BMI and thus also the better predictor of BMI than face texture. When subjected to a leave-one-out cross-validation, the correlation coefficients of BMI with the shape scores and the texture scores were 0.51 and 0.13, respectively.

Wolffhechel and colleagues [15] predicted BMI by face shape and texture using more conventional multiple regression approaches. As they require substantially more cases than variables, we performed the regressions using smaller numbers of PCs. In addition, we performed regularized regressions with all variables but different values of α, the degree of regularization. The optimal prediction of BMI by face shape was based on the first 5 PCs, yielding a correlation of BMI and the predictor of *r* = 0.66, and 0.50 after cross-validation. A regularized regression with the optimal α = 0.008 resulted in an *r* = 0.52 after cross-validation. The prediction of BMI by texture was quite unstable; the best results were given by the first PC of texture alone, yielding a correlation of 0.22. But after cross-validation, this correlation dropped to 0.03. Regularized regression required a very large α in the range of 10^9^, leading to a cross-validated correlation of about 0.18. However, this strongly regularized covariance matrix of the predictors becomes proportional to the identity matrix, and thus the whole multiple regression becomes identical to the multivariate regression of texture on shape, as we did in the first place. While combining multiple shape PCs and texture PCs did not clearly increase prediction in the multiple regression, the BMI-related shape scores and texture scores together increased the correlation to 0.61 after cross-validation.

The predictions of waist-to-hip ratio based on face shape and texture were lower than those for BMI. The correlation of WHR with the scores for WHR-related shape was 0.48 (0.31 after cross-validation) and with WHR-related texture 0.38 (0.23 after cross-validation). Together, the shape and texture scores lead to a correlation of 0.50 with WHR.

## Discussion

We studied the association of facial shape and texture with body fat by a combined geometric morphometric image analysis. While the shape of the face was represented by the shape coordinates of 119 measurement points (landmarks), the color pattern (texture) of the face was represented by the RGB values of the pixels after the images were all standardized to the same shape. This allowed us to regress both shape and texture on BMI and WHR in order to estimate the facial features maximally associated with the two variables (maximal covariance; [37]). No variable reduction for the inherently multivariate data is necessary for this regression approach.

In a similar attempt, Henderson and colleagues [38] estimated the mean difference in face shape between the ten individuals with the lowest BMI and the ten ones with the highest BMI, after a reduction of shape coordinates to a set of principal components. The resulting “BMI axis” and the projections of the individual faces onto this axis to estimate “facial BMI scores” correspond to our regression axes and the resulting shape scores. Our facial shape scores had a higher correlation with BMI than that of Henderson et al. (*r* = 0.63 vs. *r* = 0.55), presumably because we used all shape coordinates instead of PCs and the full sample for estimating the axes. The facial shape features that were associated with BMI in our sample resemble the well-known pattern: a higher BMI is associated with a relatively wider face with a rounder lower face outline, a wider nose, and lower eyebrows. Virtually the same shape features were associated with WHR.

We could further show that women with higher BMI have a brighter and more reddish facial skin color. The same pattern was found for WHR. Löffler and colleagues [14] showed that obese individuals (BMI > 30) have increased transepidermal water loss, skin blood flow, and skin color (red) compared to sex- and skin-type matched controls with a lower BMI. They suggested that a higher sweat gland activity and superficial blood flow—both being part of the temperature-regulating system—change the epidermal barrier and increase redness measures. In part, the influence of BMI on skin color may be mediated by blood pressure, since people with high BMI tend to have a higher systolic blood pressure than people with low BMI [39]. Another factor influencing skin coloration is diet. Higher fruit and vegetable intakes are related to a yellower appearance of the skin [35,40]. Women with higher BMI were found to consume less fruits and vegetables [40,41], which, in turn, might result in lower skin yellowness.

Perceived health is typically inferred from periorbital luminance and cheek redness, as well as by global skin yellowness [42]. Both carotenoid and melanin coloration enhance perceived attractiveness with a stronger preference for carotenoid in female faces [12,43]. Fisher and colleagues [44] suggested that people use facial coloration to decide whether individuals with facial shapes that indicate a low BMI are in good physical condition or ill, and use this information to judge attractiveness.

The statistical prediction of BMI or WHR from facial shape and texture is challenged by the large number of variables, which typically exceeds the number of cases. As a result, within-sample correlations overestimate the out-of-sample predictability. Cross-validation offers an approach to derive more realistic estimates of prediction accuracy. Using multiple linear regression and leave-one-out cross-validation, we were able to predict 25%-27% of variation in BMI and 10% of variation in WHR by facial shape. Facial texture predicted only about 3% of variation in BMI and WHR.

An alternative approach, also referred to as soft modeling or calibration [45], is the multivariate regression of shape/texture on BMI (instead of a multiple regression of BMI on shape/texture), which requires no variable reduction and reduces over-fitting. The resulting regression coefficients are the ones used for visualizing the shape or color pattern associated with BMI/WHR (while the partial coefficients resulting from a multiple regression can be more difficult to interpret [37,46,47]). In our sample, the resulting scores (BMI- and WHR-related shape scores) allowed for a cross-validated prediction of 26% and 10% of variation in BMI and WHR, respectively, which is as efficient as the conventional regression. Facial texture scores accounted for only 10% of BMI variation and 3% of WHR variation.

Using different regression methods based on cross-validated sets of PCs, Wolffhechel et al. [15] were able to account for 23% of BMI variation by facial shape, which is in the range of our results. Strikingly, they found that facial texture predicts 27% of variation in BMI, while we could predict only about 3% after cross-validation. This difference may result from the different regression methods and sample sizes, but more likely, it results from the differences in image registration. Most of the landmarks in our study as well as that in Wolffhechel et al. [15] are semilandmarks, that is, they are placed on homologous curves (e.g., the facial outline), but their positions along this curve are arbitrary. We use the sliding landmark algorithm [23,24], which slides the semilandmarks along their curves in order to minimize “bending energy”–a measure of local shape deformation [27]–between the individuals’ landmarks and their sample average. This is the same quantity that is minimized by the TPS interpolation, which we used for image warping [30]. Perhaps, this combined semilandmark alignment and image warping is more efficient in removing shape information from the registered images than the linear warping of Tiddeman et al. [29] and the raw landmarks used by Wolffhechel and colleagues [15], but this requires further investigation. However, combining shape and texture explained only 30% of BMI variation in Wolffhechel et al. [15], which indicates a high degree of redundancy of shape and texture variables and thus supports our explanation. In our data, by contrast, the major components of shape and texture variation were only very weakly correlated. Accordingly, combining the shape and texture scores increased the explained variance to 37% for BMI and 25% for WHR.

In our sample, both BMI and WHR ratio were associated with the same patterns of facial shape and color, despite the moderate correlation of BMI and WHR. However, in both statistical approaches, BMI was better predictable than WHR. In other words: the patterns of association were the same for BMI and WHR, but the magnitudes of association differed. This indicates that facial shape and color primarily reflects total body mass and fat proportion, which is—to a minor degree—also captured by WHR, but WHR is more influenced by the distribution of fat across the body.

Our sample comprised only young adult middle-European women. Further studies are required to assess this relationship across a wide ethnic and age diversity. Also, it is unclear if our results extend linearly to women with severe underweight or obesity, because our sample covered a BMI range from 17 to 35 only. Despite the relatively small and homogeneous sample, we were able to yield interpretable facial shape and color patterns that match those of other studies. Likewise, the predictive power of shape and texture differed clearly for BMI and WHR. However, a larger sample might improve the results about the predictive value of facial texture, which was numerically relatively unstable in our analysis. Similarly, reliable insights into the association between facial shape and facial texture, which was very weak in our sample, may require a substantially larger sample.

## Supporting Information

S1 File Questionnaire. (PDF)
