# Correction: BMI and WHR Are Reflected in Female Facial Shape and Texture: A Geometric Morphometric Image Analysis

**DOI:** 10.1371/journal.pone.0172205

**Published:** 2017-02-09

**Authors:** The PLOS ONE Journal Staff

The following information is missing from the Funding section: This study was supported by the Faculty of Life Sciences, University of Vienna (Emerging field "Comparative Human Life History: A Multilevel Approach"; IP 547012) to PM and KS, and the Austrian Science Fund (FWF P29397) to PM and SW.

In the Author Contributions section, all four authors should be listed as contributing to the methodology (CM SW KS PM).

The publisher apologizes for these errors.
