# Supplementary material for: BMI and WHR Are Reflected in Female Facial Shape and Texture: A Geometric Morphometric Image Analysis
Source: PLoS One. 2017 Jan 4;12(1):e0169336. doi: 10.1371/journal.pone.0169336 (PMC5215758; doi:10.1371/journal.pone.0169336)
Supplement: S1 File — (PDF) [file pone.0169336.s001.pdf]

Datenblatt

Teilnehmer-Nr.:

Initialen:

Datum der Datenaufnahme: Uhrzeit: Ort:

Geburtsdatum:

Geschlecht: ❒ männlich ❒ weiblich

Körperhöhe (cm): Taille (cm): Hüfte (cm):

Körpergewicht (kg):

	2D (Zeigefinger, mm)		4D (Ringfinger, mm)	
Linke Hand				
Rechte Hand				

Händigkeit: ❒ RechtshänderIn ❒ LinkshänderIn

Herkunftsland der Mutter:

Herkunftsland des Vaters:

Geburtskomplikationen, Verletzungen oder Operationen im Gesicht, kieferorthopädische oder kieferchirurgische Maßnahmen (z.B. fixe Zahnspange):

Verletzungen der oberen Extremität und sonstige Erkrankungen
(Reynaud's syndrome, cardiovascular disorder, fainting,seizures, or frostbite, or any current fractures, open cuts, or open sores on their hands or arm):

	Links<br>Verletzung	Wann	Rechts<br>Verletzung	Wann
1D				
2D				
3D				
4D				
5D				
Mittelhand, Handwurzel				
Sonstiges				

Wann: Vor wie vielen Jahren fand die Verletzung statt? Wochen, Monate in Jahre umrechnen.

Frühere oder derzeitige Einnahme von Hormonpräparaten, Medikamenten:

Erster Tag der letzten Menstruation:

Zyklusdauer:

Gewohnheiten Sonnen & Solarium

Wie oft sind Sie im letzten Jahr ins Solarium gegangen?

Seit wie vielen Jahren gehen Sie regelmäßig ins Solarium?

Wie oft haben Sie im letzten Jahr Selbstbräuner benutzt?

Seit wie vielen Jahren benutzen Sie regelmäßig Selbstbräuner?

Für wie viele Stunden Sonnenbaden Sie während der Urlaubszeit?

❑ nie ❑ weniger als 2 Stunden ❑ mehr als 2 Stunden

Sind Sie während Ihres Berufes der Sonne ausgesetzt?

❑ nein ❑ zweitweise ❑ den ganzen Tag

Rauchen

Wieviele Zigaretten rauchen Sie im Durchschnitt?

Anzahl pro Tag: ____________________

oder Anzahl pro Woche: _____________

Wieviele Zigarren, Zigarillos etc. rauchen sie im Durchschnitt?

Anzahl pro Tag: ____________________

oder Anzahl pro Woche: _____________

Alkohol

Wie oft nehmen Sie alkoholische Getränke zu sich?

❒ nie ❒ monatlich oder weniger

❒ 2–3mal pro Woche ❒ 4mal oder mehr/Woche

Wie viele alkoholische Getränke nehmen Sie an einem typischen Tag zu sich, wenn sie Alkohol trinken?

Anzahl: _______________

Wie oft im letzten Jahr konnten Sie aufgrund von Alkohol am darauffolgenden Tag das nicht tun, was Sie eigentlich tun wollten?

❒ nie ❒ weniger als einmal/Monat ❒ monatlich ❒ wöchentlich

Haben Sie heute Alkohol konsumiert?

❒ ja ❒ nein

DANKE FÜR IHRE MITARBEIT!

Bemerkungen:

Data sheet

universität wien

ID:

Initials:

Date of data collection: Time: Place:

Date of birth:

Sex: ❑ male ❑ female

Body height (cm): Waist (cm): Hip (cm):

Body weight (kg):

	2D (index finger, mm)		4D (ring finger, mm)	
Left hand				
Right hand				

Handedness: ❑ right handed ❑ left handed

Mother’s country of origin:

Father’s country of origin:

Birth complications, injuries or surgeries of the face, orthodontic treatment (e. g. fixed braces):

Injuries of the upper extremities and other diseases
(Reynaud's syndrome, cardiovascular disorder, fainting,seizures, or frostbite, or any current fractures, open cuts, or open sores on their hands or arm):

	Left Injuries	When	Right Injuries	When
1D				
2D				
3D				
4D				
5D				
Metacarpus, carpus				
Other				

When: How many years ago did the injury take place? Express weeks and months in years.

Earlier or current use of hormone drugs, medicines:

First day of last period:

Cycle duration:

Habits Sun & Tanning both

How many times have you been in a tanning both last year?

How many years have you been to the tanning both regularly?

How many times have you used self-tanning lotion last year?

How many years have you been using self-tanning lotion regularly?

How many hours are you sunbathing on vacation?

❐ never ❐ less than 2 hours ❐ more than 2 hours

Are you exposed to the sun during work?

❐ never ❐ sometimes ❐ the whole day

Smoking

How many cigarettes are you smoking on average?

Number per day: ____________________

or number per week: _____________

How many cigars, small cigars etc. are you smoking on average?

Number per day: ____________________

or number per week: _____________

Alcohol

How often do you drink alcoholic beverages?

❐ never ❐ monthly or less
❐ 2-3 times per week ❐ 4 times per week or more

How many alcoholic drinks do you drink on a typical day when you drink alcohol?

Number: ________________

How many times last year were you not able to do what you wanted to do, because of drinking alcohol the day before?

❐ never ❐ less than once per month ❐ monthly ❐ weekly

Did you drink alcohol today?

❐ yes ❐ no

THANK YOU FOR YOUR COOPERATION!

Notes:
